# The patent persistent fibrous tract in an adolescent person who presented with disconnected lumboperitoneal shunt: a case report

**DOI:** 10.1186/2045-8118-12-S1-P56

**Published:** 2015-09-18

**Authors:** Kei Yamashiro, Kiyoshi Takagi, Yasuhiro Yamada, Daisuke Suyama, Tsukasa Kawase, Yoko Kato

**Affiliations:** 1 Fujita Health University Banbuntane HotokukaiHospital, Japan; 2 Chiba Kashiwa Tanaka Hospital, Japan; 3 Fuchu Keijinkai Hospita, Japan

## Introduction

Disconnection of proximal or distal catheter is seen more frequently in the complication of the lumboperitoneal (LP) shunt. The literatures didn't establish whether the disconnected LP shunt can be allow passage of cerebrospinal fluid (CSF) though the disconnected portion. We present a case of young patient with disconnected LP shunt who reported patent persistent fibrous tract around the tube shunt.

## Case illustration

A 17-year-old man was performed LP shunt at 12 year-old due to pseudomeningocele from suboccipital decompression in the Arnold-Chiari type I malformation. LP shunt revision was performed 8 months after because of shunt malfunction. However, he complained about low back pain after revision. Radiographic studies demonstrated the proximal catheter disconnected and CSF couldn't aspirate from the flushing device. The LP shunt removal was performed and subcutaneous mass from CSF collection was observed after the closing of proximal fibrous tract by tabasco-bag suture. The patient complained about severe headache after the procedure. The lumbar puncture revealed the high pressure of 40 cmH2O. The LP shunt emergency was done then the patient turned to dramatically disappear of severe headache.

## Conclusions

Even if the shuntography cannot be performed, the clinical course can be strongly indicated that the fibrous tract remained patent after the shunt has been proved for disconnection. LP shunt operation is popular for hydrocephalus treatment and the proximal catheter disconnection is frequently found. A shuntography can identify functioning of shunt and guide management, we should perform shuntography before removal the shunt.

